# Educational Differences in Smoking among Adolescents in Germany: What is the Role of Parental and Adolescent Education Levels and Intergenerational Educational Mobility?

**DOI:** 10.3390/ijerph10073015

**Published:** 2013-07-19

**Authors:** Benjamin Kuntz, Thomas Lampert

**Affiliations:** Department of Epidemiology and Health Monitoring, Robert Koch Institute, General-Pape-Strasse 62-66, Berlin 12101, Germany; E-Mail: t.lampert@rki.de

**Keywords:** adolescence, smoking, tobacco, education, socioeconomic status, social mobility, life course, health inequalities, KiGGS, Germany

## Abstract

*Background*: Adolescence is the period in which smoking onset usually occurs and the course for future socioeconomic status (SES) is set. However, because of the transitional nature of adolescence, it is questionable whether health inequalities are best measured by indicators of parental SES or rather by indicators of the adolescents’ own developing SES. We examine the independent effects of parental and adolescent education and intergenerational educational mobility on adolescent smoking behaviour while controlling for differences in parental and close friends’ smoking behaviour. *Methods*: The study is based on data from a subsample (12–17 years, *n* = 5,053) of the nationally representative German Health Interview and Examination Survey for Children and Adolescents (KiGGS). Participants reported their education level as well as their personal and close friends’ smoking behaviour. Information on parental education and smoking behaviour was obtained via parent interviews. Adolescent and parental education data were dichotomized (low/high), leading to four categories of intergenerational educational mobility: stable high, potentially upwardly mobile, potentially downwardly mobile, and stable low. *Results*: After adjustment for parental and close friends’ smoking behaviour, adolescent smoking habits were strongly related to their personal education level, but not that of their parents. Among boys, both stable low and downwardly mobile adolescents had a 2.7-fold increased risk of being a smoker compared with peers with a stable high education. Among girls, only those with a stable low education had a 2.2-fold increased risk of smoking. Among both genders, educational upward mobility was associated with significantly lower smoking rates compared with peers with a stable low education (boys: OR 0.32; 95% CI 0.20–0.53; girls: OR 0.52; 95% CI 0.37–0.73). *Conclusions*: Our results show that the risk of an adolescent smoking is influenced by their own education level rather than that of their parents. Educational upward mobility seems to be protective against becoming a smoker in youth. Boys who experience downward mobility tend to have a significantly higher inclination to smoke than their peers with a stable high education. These findings illustrate the potential public health benefits of investments in education and help identify high-risk groups for smoking onset.

## 1. Introduction

Tobacco smoking has been identified as the most important source of preventable morbidity and premature death and the primary cause of health inequalities between socioeconomic groups in most high-income countries [1,2]. Lowering overall rates of tobacco use and reducing socioeconomic inequalities in smoking are therefore two high-priority public health goals. Because a vast majority (>80%) of adult smokers report having started smoking before 18 years of age, smoking has been referred to as a “paediatric epidemic” [3,4]. For this reason, many prevention strategies mainly focus on children and adolescents [5,6,7]. 

Although it seems evident that a lower socioeconomic status (SES) in adulthood relates to a higher smoking prevalence [8,9], the relationship between SES and smoking in adolescence is less clear. While some studies have shown considerable socioeconomic inequalities in smoking behaviour among adolescents [10,11,12], others found only weak, non-significant or even inverse associations [13,14,15,16]. In a recently published review, 15 (71%) of 21 included studies found at least some support for a negative association between SES and smoking in adolescence (higher smoking rates in lower SES groups), while only three (14%) studies indicated a positive relationship in at least one subsample [17].

Mixed findings regarding the relationship between SES and adolescent smoking behaviour seem to support a phenomenon that has been labelled “a process of equalisation” by West and others [18,19]. The term ‘equalisation’ refers to the observation that social inequalities in health and health behaviour are only slightly pronounced during the developmental stage of adolescence whereas they are usually marked in child- and adulthood. An explanation for this finding is given by the characteristics of adolescence itself (*i.e.*, the growing importance of the school environment, peers and youth culture), which affect adolescents of all SES groups in a similar way and may therefore counteract familial and social background influences [20]. Critics argue that an overly simplistic interpretation of equalisation theory could be misleading [20,21]. Instead of simply highlighting the fact that parental SES seems to be only modestly associated with various health and health behaviour outcomes, the role of alternative indicators of adolescent SES should be further studied.

In most relevant studies, adolescent SES was measured by parental characteristics such as parental education, occupational position or family income. However, without neglecting the powerful impact of parental SES, indicators such as adolescent educational aspiration, educational achievement or the type of school attended may more accurately describe adolescents’ current and future SES [22,23,24]. Studies that directly compared the impact of various SES measures on adolescent smoking behaviour suggest that parental SES indicators seem to be less strongly associated with adolescent smoking behaviour than are adolescent SES indicators [11,13,15,23,24,25,26,27,28,29,30]. Because parental and adolescent SES within a family are somewhat correlated, e.g., through intergenerational educational transmission, they should both be included in multivariate analyses to test whether both SES indicators independently relate to adolescent smoking behaviour. The significance of this method has been demonstrated in a study from Finland. In a sample of 18- to 29-year-olds, parental education was significantly associated with young adults’ daily smoking behaviour, but the effect appeared to be largely mediated by their own education level [31].

Bringing together information on parental and adolescent SES allows us to simultaneously study the effect of the accumulation of socioeconomic conditions as well as the impact of intergenerational social mobility. An accumulation effect would suggest that health consequences of social disadvantage/advantage tend to amplify if the same SES persists for more than one period in life [32,33]. Research on intergenerational social mobility has shown that upward mobility relates to an improvement in health and that downward social mobility is often accompanied by a worsening in health [34,35,36,37]. Most studies that analysed the association between intergenerational social mobility and smoking reported lower smoking rates for upwardly mobile groups and higher smoking rates for downwardly mobile groups compared with the stable SES groups of origin [15,26,35,38,39,40,41,42,43].

A major limitation of the current literature is that most studies explored the relationship between different SES measures and adolescent smoking behaviour only at a bivariate level and failed to include possibly confounding/mediating factors [10]. Parental and close friends’ smoking behaviour are amongst the most important determinants of adolescent smoking and are both also inversely associated with adolescent SES [5,15,44,45,46]. This raises the question of whether the fact that smoking is usually more prevalent among low-SES adolescents could be partly or fully mediated by the smoking behaviour of significant others.

**Figure 1:**
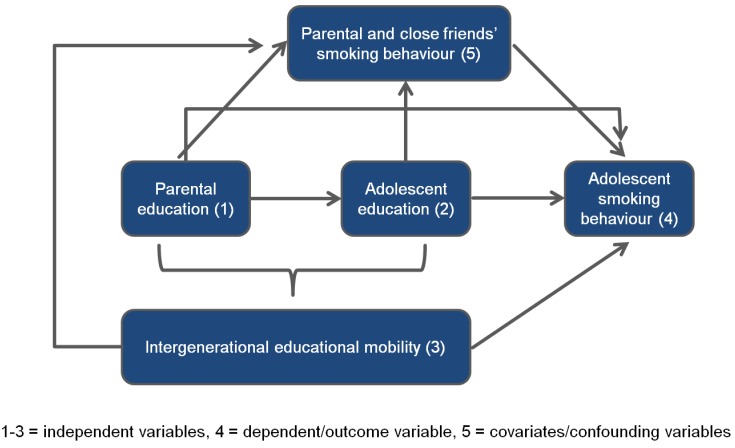
Model of the association between different educational measures and adolescent smoking behaviour allowing for parental and close friends’ smoking behaviour.

In summary, the existing literature on socioeconomic inequalities in smoking lacks studies (1) estimating the relative contribution of both parental and adolescent SES, (2) providing current and representative findings on the influence of intergenerational social mobility, and (3) controlling for socioeconomic differences in parental and peer smoking behaviour—two important determinants of adolescent smoking. Against this background, the aim of the present study was to examine whether the education levels of the parents as well as of the adolescents themselves are independently associated with adolescent smoking behaviour. Based on these two dimensions (parent education and adolescent education), we further explored how intergenerational educational mobility relates to adolescent smoking behaviour. Finally, we investigated whether the link between the three education variables and adolescent smoking habits persists after taking differences in parental and peer smoking behaviour into account. These primary research questions are illustrated in Figure 1.

## 2. Methods

### 2.1. Study Population

The data used for the following analyses were derived from the German Health Interview and Examination Survey for Children and Adolescents (KiGGS), a cross-sectional study conducted by the Robert Koch Institute from May 2003 to May 2006 [47,48]. The aim of this nationwide interview and examination survey was to collect comprehensive data on the health status of children and adolescents aged 0 to 17 years. Participants were enrolled in two steps: first, 167 nationally representative study locations (sample points) were selected; second, participants were randomly selected from the official registers of local residents. The final study population included 17,641 children and adolescents; an overall response rate of 66.6%. Study instruments were physical examinations and tests, a computer-assisted personal interview performed by study physicians, a wide range of blood and urine testing, and paper-based questionnaires. Questioning took place using a questionnaire filled in by parents, and additionally by children from the age of 11 upwards. The survey was approved by the Federal Office for Data Protection and by the Ethics Committee of Charité—University Medicine Berlin. Each parent and participant gave informed written consent before enrolment in the survey. The study protocol, including details of the sampling procedure, the execution and procedure of the study, data management, quality assurance and the inclusion of migrants has been published previously elsewhere [48].

Germany is known for its early selection in different tracks of the secondary school system [49]. For the following analyses, only data of adolescents aged 12 to 17 years will be used because almost all children at this age attend different tracks of secondary education. From the original 5,755 participants, 702 (12.2%) were excluded because of missing data on parental education, the adolescent’s educational level or the adolescent’s smoking behaviour. Table 1 contains the distribution of primary sociodemographic variables.

**Table 1:** Sociodemographic characteristics of the KiGGS sample (2003–2006) participants aged 12 to 17 years, by sex (*n* = 5,053).^ †^

	Boys (%)	Girls (%)	Total (%)
*Age in years: Mean (SD)*	14.8 (1.66)	14.9 (1.72)	14.8 (1.69)
*Sex*	2,593 (51.4)	2,460 (48.6)	5,053 (100.0)
*Region of residence*			
Newly-formed German states (incl. Berlin)	845 (19.2)	846 (19.2)	1,691 (19.2)
Old West German states	1,748 (80.8)	1,614 (80.8)	3,362 (80.8)
*Immigration background*			
Yes	360 (15.5)	341 (15.9)	701 (15.7)
No	2,233 (84.5)	2,119 (84.1)	4,352 (84.3)
*Parental education*			
High	970 (38.5)	893 (37.6)	1,863 (38.0)
Low	1,623 (61.5)	1,567 (62.4)	3,190 (62.0)
*Adolescent education*			
High	848 (33.2)	1,044 (42.1)	1,892 (37.5)
Low	1,745 (66.8)	1,416 (57.9)	3,161 (62.5)
*Intergenerational educational mobility*			
Stable high	539 (21.4)	573 (24.2)	1,112 (22.7)
Potentially upwardly mobile	309 (11.8)	471 (17.9)	780 (14.8)
Potentially downwardly mobile	431 (17.1)	320 (13.4)	751 (15.3)
Stable low	1,314 (49.8)	1,096 (44.5)	2,410 (47.2)

^†^ Percentages based on weighted data; extrapolated to the residential population of Germany (0–17 years) on 31 December 2004 (without missing data).

### 2.2. Measures

#### 2.2.1. Smoking

Adolescents were asked whether they currently smoked; smokers were asked to indicate how often they currently smoked. In our analyses, we differentiated between those who report being non-smokers and those who smoke occasionally or daily [30]. Close friends’ smoking behaviour was captured by a simple question within the participant questionnaire: “Do any of your friends smoke?” (Response categories: “yes”, “no”). Participants were encouraged to think about friends whom they considered important to them when answering this question. Information about parental smoking behaviour was obtained via parent interviews. Parental smoking, for the purposes of this study, implies occasional or daily smoking by at least one parent [5].

#### 2.2.2. Parental and Adolescent Education Levels and Intergenerational Educational Mobility

Parental education was classified according to the highest level achieved by either parent. Each adolescent’s educational status was based upon the school type they were actually attending. For adolescents who had already left school, the highest educational level achieved was used. In Germany, four school types exist at the secondary level, which offer education programs of varying length, depth and emphasis: the most basic type is secondary general school (Hauptschule), followed by the relatively more advanced intermediate school (Realschule), and the most advanced grammar school (Gymnasium), which leads to the examination that qualifies for university education (Abitur). The fourth school type is comprehensive school (Gesamtschule). This school type does not fit completely into the hierarchical system because it offers options for all three 'tracks' above [13,50]. 

For the following analyses, both parental and adolescent educational levels were dichotomized. Relatively high parental education means that at least one parent achieved a general qualification for university entrance. The ‘high’ category of adolescent education contains all adolescents who attended grammar schools. Intergenerational educational mobility was defined by combining the dichotomized categories of parental and adolescent education. The four resultant categories were: stable high (parents and the adolescent have high education), potentially upwardly mobile (adolescent’s education was higher than parents’ education), potentially downwardly mobile (adolescent’s education was lower than parents’ education), and stable low (parents and the adolescent have low education). We added the word “potentially” to the intergenerational educational mobility categories because most of the adolescents aged 12 to 17 years have not yet ended their educational career and future changes of school type are possible [51].

#### 2.2.3. Covariates

Together with parental and close friends’ smoking behaviour, we considered the following covariates in the multivariate analysis: age, region of residence (old West German states/newly formed German states, including Berlin) and immigration background. Adolescents were defined to have an immigration background (two-sided) if the adolescents themselves immigrated to Germany, both parents were not born in Germany, or both parents immigrated to Germany or had no German citizenship [52].

### 2.3. Statistical Analysis

We performed all statistical analyses using the software package SPSS 20.0 and weighted data to obtain results representative of the reference population. The weighting factor was chosen to adapt the data to the age, sex and regional residence distribution of the residential population aged 0–17 years on 31 December 2004, based on the population statistics of the Federal Statistical Office [47]. In addition to frequency analyses, we report the results of binary logistic regressions (odds ratio [OR], 95% confidence intervals [95% CI]) using the SPSS 20 procedure for complex samples that allows for the chosen sampling method in KiGGS. We differentiated between boys and girls in all our analyses to detect possible gender differences.

## 3. Results

**Figure 2:**
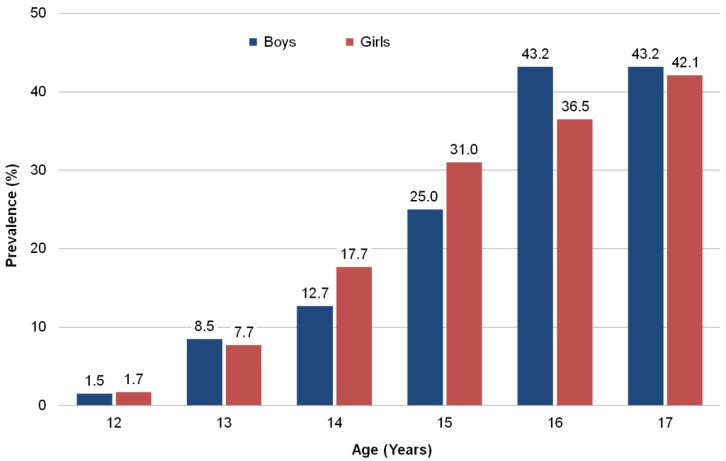
Smoking prevalence by gender and age among 12- to 17-year-old adolescents in Germany (KiGGS 2003–2006; *n* = 5,685).

According to KiGGS statistics, nearly one-quarter of 12- to 17-year-olds in Germany smoke tobacco (boys: 23.2%; girls: 23.5%). The percentage of adolescents who smoke increases with age; while smoking rates are almost negligible among 12-year-olds, more than 40% of boys and girls are smokers by age 17 (Figure 2).

### 3.1. Parental and Adolescent Education Levels and Smoking

Table 2 shows that smoking rates are negatively associated with both parental and adolescent education levels. Higher parental and adolescent education levels were linked to lower adolescent smoking rates compared with having lower parental and adolescent education levels. This also applies for parental and close friends’ smoking behaviour, meaning that adolescents from highly educated families and adolescents attending grammar schools less often had parents and close friends who smoke compared with adolescents from lower-educated families and adolescents attending lower-level secondary schools. Because parental and peer smoking behaviours are among the most important determinants of adolescent smoking, further analyses adjusted for these confounding variables to detect the unbiased effects of the different educational measures. 

Multivariate analyses show that personal (adolescent) education level had a strong and consistent impact on smoking behaviour (Table 3, Model 1). While adjustment for parental education only slightly affected the results (Model 2), further adjustment for parental and close friends’ smoking behaviour substantially lowered the odds of smoking among low-educated adolescents (Model 3). However, the fully adjusted ORs were still highly significant (boys: OR 2.85; 95% CI 2.00–4.07; girls: OR 1.76; 95% CI 1.34–2.32). Although the results for both genders are in the same direction, stronger differences regarding the impact of personal educational level on smoking behaviour occur among boys.

**Table 2:** Adolescent, parental and close friends’ smoking prevalence by education levels and intergenerational educational mobility.

	Boys (*n* = 2,593)		Girls (*n* = 2,460)	
	%	95% CI	%	95% CI
**Adolescent smoking prevalence**				
*Parental education*				
High	18.0	(15.3–21.0)	16.1	(13.7–18.8)
Low	23.2	(20.9–25.7)	25.1	(22.6–27.7)
*Adolescent education*				
High	11.9	(9.4–15.0)	16.3	(14.2–18.7)
Low	25.8	(23.4–28.4)	25.6	(23.0–28.4)
*Intergenerational educational mobility*				
Stable high	12.3	(9.4–16.0)	14.3	(11.5–17.6)
Potentially upwardly mobile	11.0	(7.9–15.3)	19.0	(15.7–22.7)
Potentially downwardly mobile	25.1	(21.0–29.7)	19.2	(14.7–24.7)
Stable low	26.1	(23.3–29.1)	27.5	(24.5–30.9)
**Parental smoking prevalence**				
*Parental education*				
High	41.5	(38.3–44.8)	40.5	(36.6–44.5)
Low	56.1	(53.1–59.1)	56.3	(53.2–59.3)
*Adolescent education*				
High	37.3	(34.1–40.7)	41.3	(37.4–45.3)
Low	57.0	(54.3–59.8)	56.9	(53.6–60.2)
*Intergenerational educational mobility*				
Stable high	33.3	(29.0–37.8)	36.8	(31.7–42.2)
Potentially upwardly mobile	44.6	(38.9–50.5)	47.4	(42.2–52.6)
Potentially downwardly mobile	51.8	(47.1–56.5)	47.0	(40.9–53.2)
Stable low	58.8	(55.4–62.2)	59.9	(56.2–63.4)
**Close friends’ smoking prevalence**				
*Parental education*				
High	45.3	(41.8–48.8)	45.2	(41.2–49.3)
Low	52.1	(49.3–55.0)	56.3	(53.5–59.1)
*Adolescent education*				
High	41.1	(37.4–44.9)	45.1	(41.7–48.6)
Low	53.6	(50.9–56.3)	57.3	(54.5–60.0)
*Intergenerational educational mobility*				
Stable high	40.6	(36.0–45.4)	41.7	(36.6–47.0)
Potentially upwardly mobile	42.1	(36.1–48.3)	49.6	(44.7–54.5)
Potentially downwardly mobile	51.2	(46.2–56.1)	51.4	(45.1–57.7)
Stable low	54.5	(51.2–57.5)	59.1	(55.9–62.2)

Converse results were found for the impact of parental education on adolescent smoking behaviour. After adjustment for age, region of residence and immigration background, low parental education was associated with a significantly higher risk of smoking among both genders (Model 1). If personal educational level is taken into account, the significant effect disappears completely among boys and drops by half among girls (Model 2). Further adjustment for parental and close friends’ smoking behaviour also leads to non-significant results among girls (Model 3).

**Table 3:** Logistic regression analyses of parental and adolescent education levels and smoking among 12- to 17-year-olds.

		Model 1 ^a^		Model 2 ^b^		Model 3 ^c^	
		OR	95% CI	OR	95% CI	OR	95% CI
*Boys (n = 2,593)*							
Parental education	High	Ref.		Ref.		Ref.	
	Low	1.53 ***	1.20–1.96	1.02	0.80–1.28	0.96	0.74–1.25
Adolescent education	High	Ref.		Ref.		Ref.	
	Low	3.57 ***	2.54–5.02	3.55 ***	2.55–4.95	2.85 ***	2.00–4.07
*Girls (n = 2,460)*							
Parental education	High	Ref.		Ref.		Ref.	
	Low	1.97 ***	1.52–2.55	1.51 **	1.16–1.97	1.31	1.00–1.72
Adolescent education	High	Ref.		Ref.		Ref.	
	Low	2.52 ***	1.94–3.27	2.21 ***	1.70–2.85	1.76 ***	1.34–2.32

^a^ Adjusted for age, region of residence and immigration background; ^b^ Model 1 plus mutual adjustment for parental and adolescent education levels; ^c^ Model 2 plus adjustment for parental and close friends’ smoking behaviour; * *p* < 0.05, **p < 0.01, ***p < 0.001.

### 3.2. Intergenerational Educational Mobility and Smoking

Smoking rates also vary by intergenerational educational mobility. Adolescents with a stable high education show the lowest smoking rates, whereas adolescents with a stable low education reveal the highest inclination towards smoking (Table 2). On the one hand, the smoking prevalence of boys who were classified as upwardly mobile is comparable to that of their peers with a stable high education. On the other hand, boys who were classified as downwardly mobile smoke about as much as their peers with a stable low education. Among girls, smoking rates of upwardly mobile and downwardly mobile adolescents do not differ and are in between the smoking rates of adolescents with a stable high or stable low educational status. Similar results were obtained when intergenerational educational mobility was linked to parental and close friends’ smoking behaviour (Table 2).

Multivariate analyses reveal that compared with adolescents with a stable high education, those with a stable low education had a significantly higher risk of smoking, even after adjustment for parental and close friends’ smoking behaviour (boys: OR 2.67; 95% CI 1.76–4.04; girls: OR 2.22; 95% CI 1.54–3.20) (Table 4, Model 2). Educational downward mobility was associated with a significantly higher inclination towards smoking among boys (OR 2.67; 95% CI 1.73–4.11), whereas the OR for girls was no longer significant after adjusting for educational differences in parental and close friends’ smoking behaviour (OR 1.53; 95% CI 0.94–2.49). Educational upward mobility, however, did not relate to an increased risk of smoking. If adolescents with a stable low education are taken as the reference group (OR = 1), potentially upwardly mobile adolescents were less likely to smoke (boys: OR 0.32; 95% CI 0.20–0.53; girls: OR 0.52; 95% CI 0.37–0.73; results are not shown in the table).

**Table 4:** Logistic regression analyses of intergenerational educational mobility and smoking among 12- to 17-year-olds.

	Model 1 ^a^		Model 2 ^b^	
	OR	95% CI	OR	95% CI
*Boys (n = 2,593)*				
Stable high	Ref.		Ref.	
Potentially upwardly mobile	0.86	0.56–1.32	0.86	0.55–1.35
Potentially downwardly mobile	3.21 ***	2.18–4.73	2.67 ***	1.73–4.11
Stable low	3.46 ***	2.34–5.10	2.67 ***	1.76–4.04
*Girls (n = 2,460)*				
Stable high	Ref.		Ref.	
Potentially upwardly mobile	1.35	0.93–1.95	1.16	0.78–1.73
Potentially downwardly mobile	1.93 **	1.21–3.08	1.53	0.94–2.49
Stable low	3.22 ***	2.26–4.58	2.22 ***	1.54–3.20

^a^ Adjusted for age, region of residence and immigration background; ^b^ Model 1 plus adjustment for parental and close friends’ smoking behaviour; *p < 0.05, **p < 0.01, ***p < 0.001.

## 4. Discussion

The present study found distinct educational differences in adolescent smoking behaviour among a representative sample of 12- to 17-year-olds in Germany. Having highly educated parents and attending more privileged secondary school types (Gymnasien) were both related to lower smoking prevalence. After mutual adjustment for parental and own education, personal education level turned out to be a stronger predictor of adolescent smoking than parental education level, with the latter having mainly indirect effects on adolescent smoking behaviour through intergenerational educational transmission [13,28]. Beyond that, intergenerational educational mobility was significantly associated with adolescent smoking behaviour. Compared with adolescents with a stable low education, those who were upwardly mobile had a significantly lower risk of smoking. Conversely, educational downward mobility was associated with a significantly higher risk of smoking compared with the “stable high” education category, at least among boys. Differences in parental and close friends’ smoking behaviour partly mediated the educational differences in adolescent smoking behaviour, but did not explain the main findings.

### 4.1. The KiGGS Data in the Light of Previous Research and Temporal Trends in Adolescent Smoking Behaviour

Our results are in line with previously published national and international studies. Based on data from the German part of the “Health Behaviour in School-aged Children” study, Richter and colleagues found significant associations between the school type attended and tobacco smoking among 11- to 15-year-old schoolchildren, while family affluence and parental occupation did not substantially affect adolescent smoking behaviour [13,23]. Other studies that directly compared the impact of various SES measures consistently report that indicators of adolescent SES are more relevant for adolescent smoking behaviour than parental SES indicators [15,24,25,26,28,29,31]. Our finding that intergenerational social mobility relates to smoking behaviour is also confirmed by the literature [15,26,35,38,39,40,41,42,43]. Lower smoking rates among young people with a stable high SES and those who experienced social upward mobility, and a higher inclination towards smoking in peers with a stable low SES and those who experienced social downward mobility, have been reported in studies from Scotland [15], France [38] and Ghana [40], among others. Already published KiGGS data have shown that intergenerational educational mobility also affects adolescents’ risk of obesity [50], multiple health behaviours [53,54], behavioural problems and their use of violence [55].

Owing to rising cigarette prices and taxes, smoking bans in public places (e.g., smoke-free schools) and intensified tobacco prevention, adolescent smoking rates have substantially declined within the first decade of the 21st century [7,56]. Annual representative surveys conducted by the German Federal Centre of Health Education indicate that between 2001 and 2011, the proportion of 12- to 17-year-old smokers dropped by more than half (from 27.5% to 11.7%) [57]. Despite this positive overall trend, according to the same dataset, relative educational differences in smoking behaviour between secondary school types remained rather stable or even rose over time [57,58]. Further studies showed that relative socioeconomic differences in adolescent smoking behaviour have either persisted (e.g., Germany 1994–2002 [13]) or increased in the past decades (West of Scotland 1990–2003 [59], Denmark 1991–2006 [60] and Finland 1977–2007 [11]). While progress has been made in achieving the public health goal of lowering overall smoking rates among adolescents, efforts to reduce socioeconomic inequalities in adolescent tobacco use have not been successful so far. Therefore, our study emphasizes the necessity of developing and intensifying specific tobacco prevention measures for adolescents attending lower-level secondary schools.

### 4.2. Gender Differences

There were some striking differences between genders in our study. While parental education did not affect boys’ smoking behaviour after taking their own education level into account, low parental education remained a significant predictor of smoking behaviour among girls. After adjustment for parental and close friends’ smoking behaviour, however, this relationship disappeared. Furthermore, we found considerable gender differences regarding one aspect of intergenerational educational mobility. Downward social mobility was significantly associated with smoking behaviour among boys, but not among girls, when allowance was made for parental and close friends’ smoking behaviour. This finding is consistent with previous results reported by Glendinning *et al*. based on data from the Young People’s Leisure & Lifestyles Project in Scotland [15]. It seems to us that downwardly mobile boys react to their possibly challenging and stressful life situation with an increased inclination towards smoking, while downwardly mobile girls might be able to develop other coping strategies. Educational upward mobility, however, seems to be protective against becoming a smoker for both boys and girls.

### 4.3. Interpreting Educational Differences in Adolescent Smoking Behaviour

Various explanations have been developed to interpret educational differences in adolescent smoking behaviour. Social learning and socialization processes, whereby adolescents tend to take up practices from their immediate social environment (especially family and peers), could play an important role [46,61]. The fact that vocational secondary school students are more likely to smoke than academic students might represent effects of differential peer clusters and school-related factors such as achievement motivation and school performance [62]. Previous research concluded that peer clusters at schools with a higher likelihood of performance problems could lead into a “school-alienated” peer climate which in turn could be considered as a risk factor for health-damaging behaviours such as smoking [13,62]. However, by controlling for parental and close friends’ smoking behaviour in our study, we were able to show that only some minor part of the relationship between different educational measures and adolescent smoking behaviour was due to differences in the smoking behaviour of significant others. The fact that smoking rates are particularly low among socially upwardly mobile adolescents might be interpreted as an attempt to overcome conventional behaviour patterns of their social class of origin [35]. In contrast, Elstad concluded in a recent paper that a “higher inclination to engage in health-compromising behaviours among low-achieving adolescents may arise from more need for stress-alleviating behaviours, less interest in the future because of unpromising social prospects, adaptation to the lifestyles of future socioeconomic milieus, attempts to compensate lack of recognition in school by excelling in alternative social fields, and deliberate opposition to social authorities because of the experience of being rejected by them” [61]. Taking these points into consideration, it seems rather unlikely that smoking prevalence is significantly higher among lower-educated adolescents solely because they might have less knowledge about the detrimental effects of tobacco consumption. Hence, classic health education—merely informing about the negative health consequences of smoking – might be insufficient to combat educational differences in adolescent smoking behaviour.

### 4.4. Strengths and Limitations

KiGGS has some major advantages compared with other studies, the most obvious being its national representativeness. A further virtue is the comprehensive dataset and the fact that both adolescents and parents were independently interviewed. While other studies completely rely on information given by the adolescents, we can assume to draw conclusions from more valid and reliable information; for instance, regarding parental education and their smoking behaviour. Of course, this does not protect us from other methodological problems. Self-reported data on substance use are generally vulnerable to social desirability effects, generally leading to inaccurate, mostly lowered smoking prevalences [63]. If better-educated adolescents are more likely to underreport their smoking behaviour, educational differences in tobacco use would be overestimated [13]. However, to our knowledge, there are no studies examining if such mechanisms substantially contribute to socioeconomic differences in smoking. Furthermore, when confidentiality is ensured, as it is in KiGGS, adolescent self-reports have been found to strongly correspond with various biochemical markers of tobacco consumption [64]. 

Because our study is cross-sectional, the direction of causality and changes in health behaviour and individual secondary school careers remain uncertain. While we tend to interpret our findings within a social causation framework (education > smoking) [61], other authors have drawn opposite conclusions in line with the indirect health-related selection approach (smoking > education) [65,66]. Koivusilta *et al*. identified smoking and other health behaviours among Finnish adolescents as predictors of educational level in later life [65,66], but for us it remains rather speculative whether smoking is really able to provoke school failure or if some other common causes underlie both smoking behaviour and educational achievements instead. Temporal ordering in terms of educational transitions and smoking uptake also favours the social causation approach. In Germany, the selection into different tracks of the secondary school system takes place very early, usually around age 10 to 12 years [49]. As the KiGGS data have shown, smoking prevalence around this age is below 2%. This fact supports Elstad’s argument “that, usually, adolescents are allocated a placement in the educational differentiation before they start engaging in common unhealthy practices” [61].

A further limitation of our study is that intergenerational educational mobility must be considered as provisional because most of the participants in our study still go to school and future changes of school type are possible [15,50]. Evidence for this is given by results of the German LifE-Study, which showed that 25% of the participants of a longitudinal study ended up with a different—in most cases higher—educational level than one would have supposed by their attended school type at age 15 years [51]. In addition, the decision to dichotomize the initial educational indicators (parental education and adolescents’ own educational status) and the smoking variables can be criticised as this approach might cause some loss of information.

Because KiGGS is being continued as a longitudinal study within the framework of the nationwide health monitoring at the Robert Koch Institute, long-term data on smoking behaviour and educational achievements will soon become available [67,68]. Based on these data it will be possible to disentangle causal effects from selection mechanisms and to analyse how educational differences in adolescent smoking behaviour track into early adulthood.

## 5. Conclusions

What our study has shown is that adolescent smoking behaviour is strongly linked to their own educational level, while the effect of parental education disappears after controlling for personal education and educational differences in parental and close friends’ smoking behaviour. Another crucial finding is that intergenerational educational mobility relates to adolescent smoking behaviour in two ways: on the one hand, educational upward mobility seems to protect adolescents from becoming smokers; on the other hand, educational downward mobility seems to stimulate tobacco smoking, at least among boys. Our findings confirm previous research by Ross and Mirowsky showing “that personal educational attainment counteracts the health effects of having poorly-educated parents” [33]. Against the background of these results, future investments in education and tobacco prevention programs that focus particularly on lower-educated adolescents as well as policies that promote upward social mobility might contribute to a further decline in smoking and subsequent burden of disease. The common interpretation of equalisation theory [18,19], that adolescence is a period in life where health inequalities are marginal, absent or even reversed, might be misleading. Given the transitional character of adolescence in itself, one could rather argue that measures of adolescents’ own developing SES (e.g., educational aspirations, type of secondary school attended) are more suitable for detecting health inequalities than parental SES indicators [20,21,40,69].
